# Supplementary material for: Sodium-Glucose Cotransporter-2 Inhibitors at Discharge from Cardiology Hospitalization Department: Decoding A New Clinical Scenario
Source: J Clin Med. 2020 Aug 11;9(8):2600. doi: 10.3390/jcm9082600 (PMC7464502; doi:10.3390/jcm9082600)
Supplement: Supplementary file 1 [file jcm-09-02600-s001.pdf]

Supplementary Materials:

Table S1. Procedures during baseline assessment and follow-up (6, 12, 18 months after hospital discharge):

Procedures:	Timeline:	Description of Procedures:
Clinical characteristics	Baseline	Age, sex, race, cardiovascular risk factors, prior cardiovascular events (coronary artery disease, peripheral artery disease, cerebrovascular disease), history of heart failure, duration of type 2 diabetes, history of retinopathy, history of kidney chronic disease, cardiovascular therapies, diagnosis at cardiology discharge.
Anthropometric parameters	Baseline and follow-up	Weight, height, body mass index [Φ].
Blood pressure	Baseline and follow-up	Systolic and diastolic blood pressure.
Laboratory tests	Baseline and follow-up	Blood plasma (fasting plasma glucose (FPG), urea, creatinine, sodium, potassium, estimated glomerular filtration rate (eGFR) [φ])<br>Urine (albuminuria)
Glycemic Profile	Baseline and follow-up	FPG, Glycated hemoglobin (%), glucose-lowering therapies.
Adherence to the GLA regimen	Follow-up	Discontinuation (yes/no) and reason of discontinuation
Clinical outcomes	Follow-up	Death from any cause.<br>Cardiovascular death.<br>Cardiovascular readmission (hospitalization).
Safety outcomes	Follow-up	≥40% decrease in basal eGFR [φ] with <60 ml/min/1.73 m^2, end-stage renal disease, or death from renal cause.<br>Hospitalization for renal cause.

[Φ] The body-mass index is the weight in kilograms divided by the square of the height in meters. [φ] Estimated glomerular filtration rate (eGFR): calculated by means of the Chronic Kidney Disease Epidemiology Collaboration equation. eGFR: estimated glomerular filtration rate; FPG: fasting plasma glucose.

Table S2: Detailed list of patients who died in the propensity-score matched cohort during follow-up.

Age (Years)	Sex	Prior History	LVEF (%)	GLD Treatment	Study Group	Survival Free of Death (Days)	Type of Death	Cause of Death
81	M	Inferior MI	60	Metformin DPP4i	Non-SGLT-2i	223	CV: non-HF	ACS
86	M	Anterior MI, AF	60	Metformin	Non-SGLT-2i	31	CV: non-HF	Sudden death
74	F	Ischemic dilated	35		Non-	474	CV: HF	HF

		cardiomyopathy		Metformin DPP4i Insulin	SGLT-2i			
61	M	Anterior MI	45	Metformin GLP1a	Non-SGLT-2i	62	CV: HF	HF
70	M	Stable angina, COPD	60	Metformin	Non-SGLT-2i	18	CV non-HF	Acute pulmonary embolism
83	M	Stable angina, AF	60	Metformin	Non-SGLT-2i	42	CV non-HF	ACS
60	M	Inferior MI	45	Metformin	Non-SGLT-2i	169	Non CV	Colorectal cancer
83	F	Stable angina, COPD	60	Metformin	Non-SGLT-2i	103	Non CV	Pneumonia
83	F	Ischemic dilated cardiomyopathy	25	Metformin	Non-SGLT-2i	87	CV: HF	HF

ACS: acute coronary syndrome, AF: atrial fibrillation, COPD: chronic obstructive pulmonary disease, CV: cardiovascular; DPP4i: dipeptidyl peptidase 4 inhibitors, F: female, HF: heart failure, M: male, MI: myocardial infarction, SGLT2i: sodium-glucose cotransporter-2 inhibitors.

Table S3: Efficacy clinical outcomes for patients in the propensity-score matched cohort by groups (SGLT-2 inhibitors/non-SGLT-2 inhibitors).

**Efficacy Clinical Outcome**	**SGLT-2 Inhibitors (N=38)**	**Non-SGLT-2 Inhibitors (N=38)**	***p*-Value**
Any cause of death	0	9 (24%)	0.001
Cardiovascular death	0	7 (18%)	0.005
• Non heart failure	0	4 (11%)	0.04
• Heart failure	0	3 (8%)	0.077
Re-admission (all causes)	9 (24%)	15 (39%)	0.09
Cardiovascular Re-admission	7 (18%)	10 (26%)	0.349
• Hospitalization for myocardial infarction or ischemic stroke	6 (16%)	6 (16%)	1
• Hospitalization for heart failure	1 (2%)	4 (11%)	0.358
Re-admission for renal cause	0	2 (5%)	0.16
Combined clinical outcome *	7 (18%)	16 (42%)	0.02

* Combined clinical outcome was defined as rate of death or readmission for cardiovascular cause.
CV: cardiovascular; SGLT2: sodium-glucose cotransporter-2.

Table S4: Other clinical outcomes during follow up by groups (SGLT-2 inhibitors group and Non-SGLT-2 inhibitors group).

Efficacy Clinical Outcomes	SGLT-2 Inhibitors, Baseline	SGLT-2 Inhibitors, Follow-Up	*p*-Value	Non-SGLT-2 Inhibitors, Baseline	Non-SGLT-2 Inhibitors, Follow-Up	*p*-Value
eGFR (ml/min/1.73 m^2)	77.343 (+/- 18.148)	77.455 (+/- 21.315)	0.253	71.886 (± 21.351)	63.966 (+/- 25.29)	0.028
Systolic blood pressure (mmHg)	124.8 (+/- 16.3)	126.05 (+/-15.3)	0.731	125.19 (+/- 13.7)	127.5 (+/-15.2)	0.954
Diastolic blood pressure (mmHg)	69.9 (+/- 8.9)	70.5 (+/- 9.2)	0.766	68.6 (+/-13.15)	72.1 (+/- 11.6)	0.221
Weight (kg)	81.68 (+/-13.1)	79.1 (+/-12.4)	0.001	77.43 (+/- 17.4)	77.9 (+/- 16.4)	0.369
Glycated hemoglobin (%)	7.79 (+/-1.59)	7.20 (+/-0.94)	0.1	7.29 (+/- 1.62)	7.20 (+/-1.51)	0.818

eGFR: estimated glomerular filtration rate; SGLT-2: sodium-glucose cotransporter-2.
